# Psychometric Properties of the Greek Version of the Caring Behaviors Inventory-16

**DOI:** 10.7759/cureus.15186

**Published:** 2021-05-23

**Authors:** Victoria Alikari, Evangelos C Fradelos, Evridiki Papastavrou, Stavroula Alikakou, Sofia Zyga

**Affiliations:** 1 Department of Nursing, Faculty of Human Movement and Quality of Life Sciences, University of Peloponnese, Tripolis, GRC; 2 Department of Nursing, University of Thessali, Larissa, GRC; 3 Department of Nursing, Faculty of Health Sciences, Cyprus University of Technology, Limassol, CYP

**Keywords:** caring behaviors inventory, nurses, patients, reliability, validity, scale

## Abstract

Caring is a key component of nursing. Exploring patients' perceptions of caring behaviors is essential to providing high-quality health care. This study aimed to investigate the validity, reliability, and internal consistency of the Greek version of the Caring Behaviors Inventory-16. This descriptive cross-sectional study involved hospitalized patients from six major hospitals in Greece who completed the Caring Behaviors Inventory-16 scale. This is a self-completing questionnaire whose answers range from one to six on a Likert-type scale. The overall score ranges from 16 to 96. To study the reliability of the scale, 50 patients completed the scale twice within two weeks, and then the repeatability was tested using the Pearson’s r correlation coefficient and the intraclass correlation coefficient. Construct validity and internal consistency were tested among 180 patients. Construct validity was tested through the principal component analysis. The internal consistency was tested through Cronbach’s alpha index. The statistical analysis was performed through the IBM Statistical Package for the Social Sciences (SPSS) Statistics Version 21.0 (Armonk, NY: IBM Corp.). The level of statistical significance was set at 5%. The study was conducted in the period October-December 2019. According to the results, the average age of patients was 58 years old and 50.6% were men. The mean value of the scale was 79.31 (standard deviation ± 15.75). The principal component analysis showed that the scale is unidimensional highlighting one factor that explains 68.24% of the total variance. Questions loadings ranged from 0.575 to 0.912 on the same factor. This means that all questions measure the same structure and are strongly concentrated in the same construction. Regarding the repeatability test, no statistically significant differences were observed between the two measurements. Pearson's r coefficient was 0.82 while the intraclass correlation coefficient was 0.91 (p<0.001) and indicate the very good reliability of the scale. Cronbach’s alpha was 0.967 and indicates the excellent internal consistency of the scale. Data analysis showed that the Caring Behaviors Inventory-16 is a valid, reliable, simple, and short tool for assessing patients' perceptions of caring behaviors. Further tests are suggested to confirm the construct validity, reliability among patients, nurses, or nursing students.

## Introduction

Nursing is the basic component and the cornerstone of caring. Although a body of knowledge has been produced about caring, the concept remains unclear and elusive, difficult to define, and more difficult to measure or observe [1]. However, it manifests in day-to-day nursing practice, and most scholars are using caring behaviors as a functional term to describe and explain care. It seems that most researchers agree that care is a multidimensional phenomenon with a variety of domains. The emotional dimension of caring involves providing emotional support to the patient through the acceptance of feelings, empathy, trust, hope, and emotional warmth. The physical dimension of caring refers to activities such as care of hygienic needs, the promotion of physical comfort, and cognitively oriented interventions [2]. The ethical dimension of care includes respect and dignity of the patient’s personality, regardless of race, gender, age, socio-economic status of the patient, or the nature of the disease. The ethical component is a key element in building successful therapeutic relationships and communication between nurses and patients in order to achieve goals, such as patient information, education, and satisfaction [3].

The concept of caring differs between cultures. Health caring needs to be culturally sensitive due to the different cultural features that may conflict with each other such as expressive style, eye expression, personal space, touch, dietary preferences, and religious habits [4]. Caring presupposes the availability of the nurse, a supportive work environment, professional maturity, and a strong ethical basis [5].

Jean Watson's theory related to human caring highlights the experience of each patient in the caring process and argues that effective caring will benefit the patients’ health outcomes [6]. Patients perceive their caring based on how they feel about nurses' caring behaviors [7]. Caring attitudes are probably the greatest predictor of a positive experience of health service users. Many studies highlight its impact on satisfaction, comfort, and perception of safety by patients and family members [8]. Although caring is essential for patient satisfaction, changes in healthcare systems around the world have intensified nurses' responsibilities and workloads, making it difficult to achieve traditional nurse-patient relationships [9].

Studies on caring have focused on nurses' and students’ perceptions of what caring means [10], patients' perceptions of what is important for them to feel cared for [11], and comparisons between patients' and nurses’ perceptions of caring [12]. Studies have shown that patients' perceptions of caring may not coincide with nurses' perceptions, especially when patients and nurses come from different national or cultural backgrounds and interpret related concepts differently [7,12].

Patients' perceptions of caring are investigated with quantitative (cross-sectional) and qualitative (phenomenological) or mixed studies [13]. Quantitative studies use a variety of checklists to measure caring actions; this choice has undoubtedly advantages due to its practicality and the possibility to compare different contexts. Many instruments measure caring and many of them have been constructed to study caring behaviors as perceived both by nurses and patients [14]. Care-Q is an anonymous questionnaire that was constructed according to the Q-methodology and investigates nurses’ caring behaviors through six dimensions (“trusting relationship,” “monitors,” “accessible,” “comforts,” “anticipates,” and “follow-through”). It is the most common tool for measuring caring behaviors. A second tool described is the caring behaviors assessment tool (CBA). The CBA consists of 63 items grouped in seven domains (“humanism,” “trust,” “expression of feelings,” “teaching/learning,” “supportive environment,” “needs assistance and existential forces”). The most important state of this scale is “makes me feel someone is there if I need them”. The Nurse/Patient Caring Questionnaire (N/PCQ) is a 61-item questionnaire exploring caring behaviors in the domains of “the importance of caring,” “attributes of the nurse,” “professional vigilance,” and “interaction”. It is available for completion for both nurses and patients. The Caring Dimensions Inventory (CDI) has been used for both nurses and students and it consists of 35 actions grouped in five dimensions (“psychosocial,” “technical,” “professional,” “inappropriate,” and “unnecessary actions”) [14]. The Caring Behaviors Inventory scale (CBI) was developed based on Watson’s theory [15]. It has been revised in several versions (43 and 42-item, 24-item, and 6-item) with excellent reliability (Cronbach’s alpha 0.95-0.98) [16]. The various versions of this scale study the dimensions of “safety,” “knowledge and skills,” “respectful deference,” “connectness,” and “human presence” [16]. The CBI-24 has been validated in a sample of Cypriot and Greek nurses and patients [17]. In 2017, the third version of CBI was constructed consisting of 16 statements [18].

Given that the previous questionnaires are large in extent, this study aimed to explore the construct validity, reliability, and internal consistency of the Greek version of the CBI-16 in a sample of hospitalized patients in Greece. Also, apart from the study of the validity and reliability of the scale by Wolf et al., no other studies related to the psychometric properties of the 16-item scale in other cultures have been found [18].

## Materials and methods

This is a descriptive, cross-sectional study. To conduct the study, the following research plan was followed.

Study design 

Hospitalized patients coming from six Greek general hospitals of Athens (one hospital), Piraeus (one hospital), Peloponnese (three hospitals), and Thessaloniki (one hospital) regions had participated. The above regions are some of the most populated counties in Greece. The inclusion criteria were (a) being > 18 years old and (b) able to read and write in the Greek language. The exclusion criteria were (a) patients suffering from mental illnesses and (b) patients with severe eye problems. The questionnaires were randomly distributed to 200 patients of the above hospitals. In total, 180 questionnaires were completed (response rate of 90%). According to the literature, in studies of reliability and validity of tools for each question of the questionnaire, 10 participants are required [19]. Therefore, the number of 180 participants is sufficient as the CBI-16 consists of 16 questions. The study was conducted during the period of October-December 2019. The questionnaires were provided to patients by the researchers.

The instrument

The Caring Behaviors Inventory-16 (CBI-16) was developed in 2017 to assess perceptions of caring behaviors as perceived by nurses and patients [18]. It is an anonymous questionnaire that consists of 16 states rated on a six-point Likert scale (1= never to 6= always). The CBI-16 was developed after the revision of the CBI-42 and CBI-24 and the retention of the items. The total score can range from 16 to 96. The higher the score, the higher the perception for each caring behavior. Even the CBI-24 studies report four domains of caring (“human presence,” “professional knowledge,” “respectful deference,” and “connectness”), the CBI-16 is unidimensional [18]. Since the 16 questions of CBI-16 are contained in the Greek version of CBI-24, there was no need for translation and cultural adaptation in the Greek language [17]. The initial versions of the scale (CBI-42 and CBI-24) have been used in several studies with high internal consistency (Cronbach's alpha = 0.96) [20,21]. The CBI-16 is short, easy to be analyzed and one of the few instruments which can be used for nurses and patients facilitating the comparisons [17].

Reliability

To test the reliability, 50 patients completed the questionnaire two times, initially and two weeks after the first time. According to the literature, to test the repeatability of the scales, a period of two weeks between the first and second completion is required to take so that the participants do not remember their answers [22].

Statistical analysis

Quantitative variables were described with the mean values and standard deviations while qualitative variables were described with the absolute (N) and relative (%) frequencies. To investigate the construct validity of the scale principal component analysis was performed. The reliability of the CBI-16 was tested through the test-retest method. Pearson’s correlation coefficient and the intraclass correlation coefficient (ICC) were used to test the repeatability between the first and the second completion of the scale. The Cronbach’s alpha coefficient was used to test the internal consistency of the Greek version of the CBI-16. For the data analysis, the IBM Statistical Package for the Social Sciences (SPSS) Statistics Version 21.0 (Armonk, NY: IBM Corp.) was used while the statistical significance was set up at the level of 5%.

Ethics

Permission from the Scientific Councils of the Hospitals (General Hospital of Athens G. Gennimatas, General Hospital of Piraeus St. Panteleimon, General Hospital of Thessaloniki G.Papanikolaou, General Hospital of Korinthos, General Hospital of Kalamata, General Hospital of Kyparissia) was secured since these councils are also considered as ethics committees for the hospitals. Participants were informed about the anonymity and the volunteering of the study, that the data will be used only for research purposes and that they can withdraw from the study if they want. Also, patients signed a written consent form.

## Results

As presented in Table 1, the mean age of the patients was 58 years old (standard deviation {SD} ± 16.6), of all, 50.6% were men where the majority had high school education (45%). Most of the patients were hospitalized in clinical departments of the Internal Medicine Sector and the mean duration of hospitalization was 10.72 (SD ± 9.7) days.

**Table 1:** Patients’ demographic and clinical characteristics (Ν=180) SD: standard deviation

Demographic data	Frequency	Percentage (%)
Gender		
Female	89	49.4
Male	91	50.6
Educational status		
Primary school	54	30
High school	81	45
University	38	21.1
MSc/Ph.D	6	3.3
Sector of hospital		
Internal medicine	129	71.7
Surgical sector	50	27.8
Reason for admission		
Chronic disease	103	57.2
Acute disease	75	41.7
	Mean (SD)	Min-Max
Age (years)	58 (16.6)	19-82
Duration of hospitalization (days)	10.72 (9.7)	1-60

Construct validity

**Figure 1:**
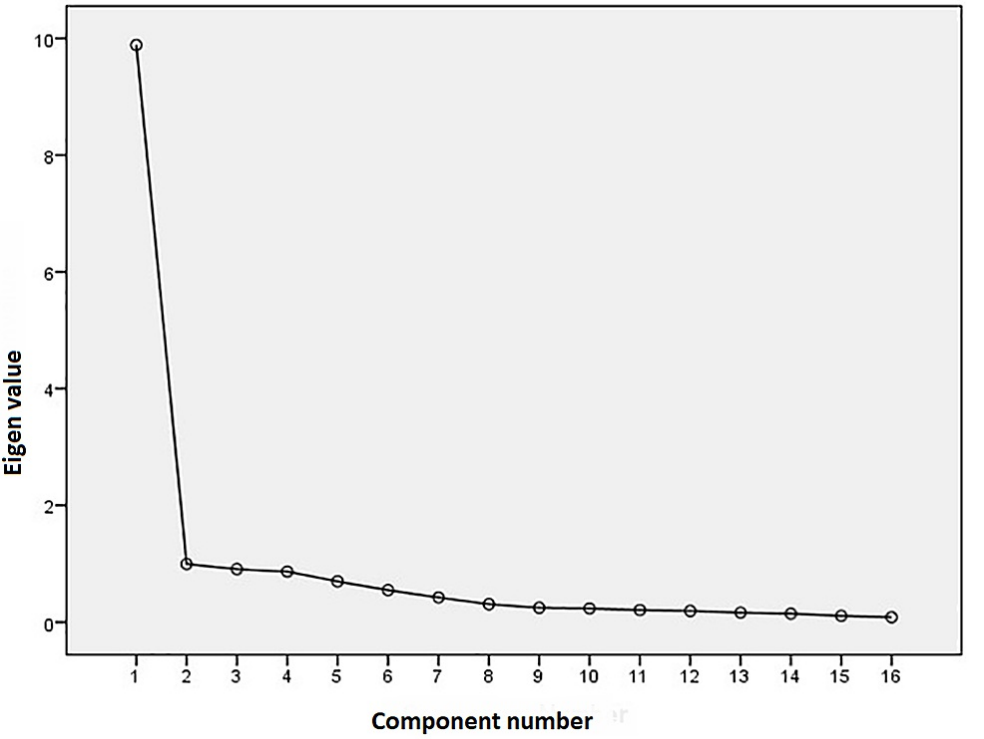
Scree plot for CBI-16 scale CBI-16: Caring Behaviors Inventory-16

The value of the Kaiser-Meyer-Olkin Index (0.941) for sampling adequacy and Bartlett's Test of Sphericity (x2 (120) = 2794.142, p<0.001) showed that there is sampling adequacy. The principal component analysis (PCA) revealed a one-component solution as it is depicted in the scree plot (Figure 1).

The PCA of CBI-16 items extracted a unique factor explaining 68.24% of the variance (Criterion Kaiser, Eigenvalue >1). The queries loaded from 0.575 (item 10) to 0.912 (items five, six, and13). The acceptable limit of the items loading is >0.40 [23]. This shows that all statements measure the same construct of caring behaviors and are strongly concentrated in this construction, showing high linking to the construction (Table 2).

**Table 2:** Items loading of the CBI-16 Kaiser-Meyer-Olkin measure of sampling adequacy=0.941; Bartlett's test of sphericity: x2 (120) = 2794.142; df=120; p<0.001; extraction method: principal component analysis. CBI-16: Caring Behaviors Inventory-16

Items	1 Component
Nurses attentively listening to me	0.827
Nurses giving instructions or teaching me	0.813
Nurses treating me as an individual	0.877
Nurses spending time with me	0.910
Nurses support me	0.912
Nurses are empathetic or identify with me	0.912
Nurses are confident with me	0.877
Νurses demonstrate professional knowledge and skill	0.610
Nurses include me in planning my care	0.753
Nurses treat my information confidentially	0.575
Nurses return to me voluntarily	0.778
Nurses talk with me	0.911
Nurses meet my stated and unstated needs	0.912
Nurses respond quickly when I call	0.858
Nurses give my treatments and medications on time	0.784
Nurses relieve my symptoms	0.810

Reliability

No significant differences existed between the two measurements. This finding indicates the stability and reliability of the scale. The ICC both at the level of total score and items separately revealed an excellent correlation between the first and the second measurement (ICC=0.991, p<0.001). Also, the Pearson correlation r in the total scale showed a strong correlation between the two measurements (r=0.82, p<0.001) (Table 3).

**Table 3:** Test-retest reliability of the CBI-16 (n=50) p<0.001 SD: standard deviation; ICC: intraclass correlation coefficient; CBI-16: Caring Behaviors Inventory-16

CBI-16 items	Mean (SD±) 1st test	Mean (SD±) 2nd test	Pearson’s r correlation	ICC
1	4.94 (1.20)	5 (1.16)	0.91	0.96
2	4.90 (1.03)	4.96 (0.99)	0.88	0.96
3	4.33 (1.03)	4.27 (1.34)	0.90	0.97
4	4.5 (1.12)	4.41 (1.16)	0.87	0.97
5	4.65 (1.18)	4.6 (1.22)	0.84	0.95
6	4.55 (1.47)	4.44 (1.54)	0.92	0.95
7	4.87 (0.97)	4.84 (0.99)	0.79	0.97
8	5.08 (1.12)	5.01 (1.15)	0.88	0.96
9	4.48 (1.31)	4.41 (1.35)	0.89	0.93
10	5.28 (1.1)	5.27 (1.09)	0.90	0.94
11	4.62 (1.32)	4.56 (1.36)	0.83	0.95
12	4.48 (1.32)	4.43 (1.3)	0.89	0.96
13	4.6 (1.19)	4.54 (1.23)	0.80	0.94
14	4.76 (1.23)	4.7 (1.25)	0.81	0.95
15	5.06 (1.11)	5.05 (1.12)	0.84	0.94
16	4.88 (1.11)	4.8 (1.23)	0.79	0.95
Total	75.98(15.4)	75.29 (15.2)	0.82	0.91

Internal consistency

The internal consistency of the CBI-16 was tested through Cronbach’s alpha Index which was found to be 0.967. This result indicates that the CBI-16 has excellent internal consistency. In addition, in the case of deletion of an item, there would be no increase in Cronbach’s alpha index. In Table 4 below, the total mean score, the means of each item, and the results of internal consistency are presented. Each item was strongly correlated to the total score (>0.3) as performed by the corrected item to total correlations (values 0.54 - 0.89). The total score was 79.31 (SD ±15.75). The highest value is pointed out in item 10 (Nurses treat my information confidentially) while the lowest in item 9 (Nurses include me in planning my care).

**Table 4:** Internal consistency of CBI-16 (N=180) SD: standard deviation; CBI-16: Caring Behaviors Inventory-16

CBI-16 items	Mean (SD±)	Min-Max	Corrected item-total correlation	Cronbach's alpha if item deleted	Cronbach's alpha
1	5.14 (1.1)		0.8	0.965	
2	5.04 (1.1)		0.78	0.965	
3	4.67 (1.42)		0.85	0.964	
4	4.75 (1.23)		0.89	0.963	
5	4.84 (1.31)		0.89	0.963	
6	4.73 (1.47)		0.89	0.963	
7	5.13 (0.99)		0.85	0.964	
8	5.32 (0.91)		0.57	0.966	
9	4.4 (1.5)		0.72	0.965	
10	5.42 (0.98)		0.54	0.966	
11	4.63 (1.35)		0.75	0.966	
12	4.73 (1.28)		0.89	0.963	
13	4.87 (1.2)		0.89	0.963	
14	5 (1.1)		0.83	0.964	
15	5.36 (0.95)		0.75	0.966	
16	5.19 (1.07)		0.77	0.965	
Total	79.31(15.75)	35-96			0.967

## Discussion

This study was carried out in six general hospitals in Greece among various patients and aimed to assess the validity and reliability of the Greek version of the Caring Behaviors Inventory-16. The psychometric properties of CBI-16 were first tested in the study of Wolf et al. in a sample of patients in Philadelphia and South Jersey, USA [18]. This is the first attempt to study the psychometric properties of the 16-item scale outside the USA. To the best of our knowledge, in this study, the CBI-16 was used for the first time among Greek patients and provides support that the tool is a simple, valid, and reliable instrument to assess perceptions of caring behaviors among Greek patients and nurses. Even the previous versions of CBI (24 and 42 items) have been used in many studies and cultures, the 16-item CBI has not been used widely [24,25]. This will probably be because this is new construction. This places limitations as no comparisons can be made with previous studies in which CBI-16 has been used.

As far as the construct validity is concerned, the PCA revealed the unidimensionality of the 16-item CBI by generating one solution explaining 68.24% of the total variance. The factor analysis of the 43-item scale revealed five dimensions (human presence, professional knowledge, respectful deference, and connectness and attentiveness to the other’s experience) [26] while the 24-items scale revealed four dimensions [17]. The unidimensionality of the scale in the current study is supported in the study of the validity of the revised version of the CBI-24 from which the short CBI-16 was produced [18]. All item loadings are high (>0.40) pointing out the conceptual relevance to Watson’s carative factors: trusting relationship (items 5, 7-10), participation in authentic teaching (item 2), being present (items 1, 3, 11, 12), loving-kindness and equanimity within a context of caring consciousness (item 6), and basic needs (items 13-16) [27]. Comparing the item loadings of our study to the item loadings of the above study of Wolf et al., it seems that most of the items showed higher loadings in the current study except items 8 (“nurses demonstrating professional knowledge and skill”) and 10 (“nurses treat my information confidentially”) [18]. In fact, these two items marked efficient but low loadings. This finding could be explained on the basis of the different cultural backgrounds of patients since nurses' professional knowledge and skills are underestimated by Greek patients while personal contact and interest are overestimated. On the contrary, item 8 was of great significance in the study using the CBI-24 among Greek nurses suggesting the divergence of views between patients and nurses [17].

The test-retest reliability of the scale was, also, acceptable since the ICC ranged from 0.93 to 0.97 and the correlation coefficient r ranged from 0.79 to 0.92. The value of Cronbach’s alpha (0.967) and the strong correlation between each item to total score indicates the excellent internal consistency of the CBI-16. This value is higher than that of the first study (0.95) [18]. Also, the highest mean (5.42) was observed in item 10 (“nurses treat my information confidentially”) while the same result was observed in a previous study where the particular item was rated 5.74 [18]. Τhis result highlights the confidentiality and integrity of nurses but also the preservation of their individuality. Similar results were presented regarding the lowest mean (Item 9, “nurses include me in planning my care”) in previous studies [17,18,28]. This finding may be due to the fact that the biomedical model still prevails and nurses usually do not include patients in the clinical decision-making process.

Strengths and limitations

A strength of this study arises from the fact that the participants came from hospitals located in large geographical regions in Greece (Attica, Peloponnese, and Thessaloniki regions). Also, patients come from various clinical wards indicating the diversity of the sample. Therefore, we can assume that the results of this study may be generalized. Additionally, this is the first study trying to check the psychometric properties of the CBI-16 in other cultures after the study of Wolf et al. [18]. Regarding the limitations, it should be considered that patients completed the questionnaires in their wards. Consequently, the presence of other persons inside the wards (doctors, attendants, and other health professionals) may have affected the answers.

## Conclusions

The con­struct validity and reliability of the Greek version of the CBI-16 are supported by patients. Therefore, the Greek version of the CBI-16 is a valid and reliable tool for assessing the perceptions of caring behaviors. Αs it is a short and simple questionnaire, it can be an alternative to other longer tools that have been used in the past, even if it should be reinforced with further tests to confirm construct validity. Also, it can be used for patients, nurses, and nursing students in order to make comparisons in future researches. Identifying tools for measuring caring and analyzing how caring affects patient outcomes can guide nursing practice and ensure that nursing caring remains a priority as nursing technical tasks.
